# Proof of Concept for the Autobiographical Memory Flexibility (MemFlex) Intervention for Posttraumatic Stress Disorder

**DOI:** 10.1177/2167702620982576

**Published:** 2021-03-31

**Authors:** Ali Reza Moradi, Maryam Piltan, Mohammad Hasan Choobin, Parviz Azadfallah, Peter Watson, Tim Dalgleish, Caitlin Hitchcock

**Affiliations:** 1 Department of Psychology, Institute for Cognitive Sciences Studies, Kharazmi University; 2 Department of Psychology, Tarbiat Modares University; 3 Department of Clinical Psychology, Kharazmi University; 4 MRC Cognition and Brain Sciences Unit, University of Cambridge; 5 Cambridgeshire and Peterborough NHS Foundation Trust, Cambridgeshire, England

**Keywords:** autobiographical memory, cognitive processes, posttraumatic stress disorder, randomized controlled trials, open data, preregistered

## Abstract

Autobiographical memory distortions are a key feature of posttraumatic stress disorder (PTSD). In this proof-of-concept randomized controlled trial (*N* = 43), we evaluated an autobiographical memory flexibility intervention, MemFlex. We aimed to determine whether the mechanism-focused intervention, which aims to improve autobiographical memory processes, may also affect other cognitive predictors of PTSD and potentially reduce PTSD symptoms in Iranian trauma survivors diagnosed with PTSD. Results indicated significant, moderate to large between-groups effect sizes in favor of MemFlex, relative to wait-list control, for the targeted cognitive mechanism of autobiographical memory flexibility and PTSD symptoms. A large, significant effect was also observed on maladaptive posttraumatic cognitions—a strong predictor of PTSD prognosis, which is a key target of high-intensity cognitive therapies for PTSD. Findings support future completion of a scaled-up trial to evaluate treatment efficacy of MemFlex for PTSD to determine whether MemFlex may offer a culturally adaptive, low-cost, low-intensity intervention able to improve cognitive mechanisms of PTSD.

Posttraumatic stress disorder (PTSD) is commonly conceptualized as a disorder of memory (Dalgleish, 2004). In addition to the defining memory-related symptoms of intrusive distressing memories and vivid reliving of the traumatic event via flashbacks (American Psychiatric Association, 2013), cognitive theories of PTSD propose that the maladaptive appraisals that drive the disorder are anchored in the memory of the trauma itself (Ehlers & Clark, 2000). It is therefore unsurprising that intervention techniques to improve the fragmented nature of a trauma memory (see Brewin et al., 1996), use of exposure to reduce distress related to the trauma memory, and revisiting the trauma memory to identify the posttraumatic appraisals that are most maladaptive for the individual are key features of the most successful treatments (e.g., trauma-focused cognitive behavioral therapy [CBT], eye movement desensitization and reprocessing [EMDR]) for PTSD (Section 1.6 in National Institute for Health and Care Excellence, 2018).

Completion of these gold-standard psychological interventions requires access to a health care professional who has received explicit training and ongoing supervision in these specialist techniques (National Institute for Health and Care Excellence, 2018). This limits access to intervention in situations in which such training may not be readily available because of a lack of experts to deliver training, a lack of resources to fund specialist training, or indeed, a lack of mental health care practitioners in general (e.g., in rural or isolated communities, active war zones). It is also common that complex cognitive-focused interventions such as trauma-focused CBT are not delivered in situations in which the individual is still exposed to danger because phenomenon such as hyperarousal to threat may actually be adaptive for the individual. Reduced access to cognitive-focused therapy poses a problem for effective treatment of PTSD because the cognitive processes targeted by these therapies are among the strongest predictors of PTSD prognosis (e.g., disjointed narratives in trauma memories and maladaptive appraisals about the event predict future symptoms over and above trauma severity and initial symptoms; see Halligan et al., 2003).

A basic-science understanding of the cognitive predictors of PTSD could help to identify mechanisms that can be targeted using (a) low-cost, low-technology, easily accessible interventions that can (b) be delivered by individuals without specialist mental health care training. Autobiographical memory for personal life experiences represents one potential cognitive mechanism that can be targeted in this way. Although the autobiographical memory of the traumatic experience is central to the perpetuation of PTSD symptoms, broader processes in autobiographical memory also uniquely predict prognosis. A reduced ability to recall specific, single-incident events (even of events that are not trauma related) has been consistently observed to predict the course of PTSD symptoms over and above initial symptom levels (Bryant et al., 2007; Harvey et al., 1998; Kleim & Ehlers, 2008). Reduced memory specificity also impairs a number of other everyday cognitive processes that support posttraumatic recovery, such as problem-solving (Goddard et al., 1996; Jing et al., 2016; McFarland et al., 2017), imagining and planning for the future (Jing et al., 2017), and maintaining social connectedness (Alea & Bluck, 2003; Beike et al., 2016). Indeed, interventions to improve autobiographical memory specificity (e.g., memory specificity training [MEST]) have been shown to also improve PTSD symptoms (Maxwell et al., 2016; Moradi et al., 2014).

However, we recently demonstrated that other autobiographical retrieval processes may also be impaired in PTSD (Piltan et al., 2021). Prior work (Hitchcock et al., 2019) has demonstrated that the ability to retrieve general memories that summarize categories of events and the ability to flexibly move between specific and general memories was also impaired in depression, in addition to the ability to retrieve specific memories. Critically, intervention to improve memory flexibility has been shown to improve depressive symptoms in clinical populations (Hitchcock et al., 2015, 2016, 2021). When evaluating the deliberate retrieval of specific memories, general memories, and flexible movement between the two memory types in trauma survivors in Iran, we demonstrated that relative to trauma-exposed and community control participants, individuals with PTSD experienced not only reduced memory specificity but also reduced memory flexibility (Piltan et al., 2021). These findings therefore suggest that reduced memory flexibility may be a transdiagnostic marker of emotional disturbance and that targeted intervention to improve not only memory specificity but also memory flexibility may be useful to people with PTSD. For example, planning for future events (e.g., purchasing a coffee) requires dynamic recombination (Addis, 2018) of generalized summaries of the past that provide a blueprint of what to expect (e.g., “the barista will ask what type of coffee I would like”) and specific episodic information (e.g., “I really enjoyed the Columbian blend last time I was at this café”). Training flexible movement between specific and general levels of memory representation may thereby enhance cognitive skills that support recovery from PTSD.

It would be particularly interesting if training autobiographical memory processes more broadly could positively affect the trauma memory or associated posttraumatic appraisals without the individual ever discussing the trauma or its meaning or deliberately retrieving the trauma memory itself. That is, we were interested in whether improving the ability to retrieve concrete, specific details of all memories would flow on to increases in the (typically poor) visual and sensory quality and temporal features of a trauma memory. Because poor quality of a trauma memory is associated with poorer prognosis (Ehlers & Clark, 2000), intervention-driven improvement in trauma memory quality may represent one potential mechanism through which autobiographical memory-based training programs help to improve PTSD (Hitchcock et al., 2017; Moradi et al., 2014). Likewise, training someone to flexibly move between specific events and generalized representations of the past may help to constrain overly generalized, negative beliefs (as suggested by the results of Hitchcock et al., 2017). Because the extrapolation of meaning attributed to the trauma (e.g., other people cannot be trusted, there is something wrong with me as a person) to the self and world more broadly is another strong predictor of prognosis, any effect of intervention on generalized posttraumatic appraisals could represent an alternate mechanism through which autobiographical memory-based training programs help to improve PTSD.

We therefore sought to evaluate the potential of a memory flexibility training (MemFlex) program in ameliorating autobiographical memory deficits and other cognitive predictors of PTSD. The MemFlex intervention is a workbook-based program that has previously proved efficacious in improving both autobiographical memory deficits and symptoms of depression (Hitchcock et al., 2016, 2018, 2021). Adaptation of MemFlex techniques has also yielded promising findings in individuals experiencing psychosis (Edwards et al., 2020). In addition to more comprehensively targeting autobiographical memory difficulties associated with PTSD, if effective, MemFlex could offer a number of benefits for improving access to PTSD treatment relative to other low-intensity cognitive interventions. The intervention uses a paper workbook and therefore does not require access to a psychotherapist, a computer, or the Internet to complete. There is a brief 30- to 45-min initial face-to-face session to orient the individual to the workbook, but this is designed to be (and has previously been effectively) delivered by someone without any mental health care training (Hitchcock et al., 2018, 2021). Furthermore, the intervention does not involve working with trauma memories or symptoms of PTSD at all. Rather, individuals simply complete cued recall tasks that aid them to retrieve positive and emotionally benign personal memories. MemFlex is therefore likely to be less distressing for an individual to complete without clinician support. The MemFlex intervention could therefore offer significant potential for improving access to cognitive-based PTSD treatment around the world.

Here we present a randomized controlled trial which sought to establish proof of concept for MemFlex as a potential treatment for PTSD. Our primary aim was (a) to determine whether MemFlex could improve deliberate retrieval of autobiographical memories (our targeted cognitive mechanism) in individuals diagnosed with PTSD and (b) to estimate the treatment effect size on PTSD symptoms to determine whether a later-phase, scaled-up trial is warranted and (c) to inform a sample-size calculation for such a trial in line with recommendations for the development of novel interventions (Craig et al., 2008; Medical Research Council, 2000). Effects were compared between the MemFlex condition and a wait-list control condition. Our secondary aim was to explore potential effects of MemFlex on other cognitive predictors of PTSD, including maladaptive posttraumatic appraisals, rumination, and quality of the trauma memory, to inform the selection of process measures for any later-phase trial.

## Method

### Trial registration

The trial methods and sample size were preregistered on clinicaltrials.gov (NCT03634709). Ethics approval was obtained from the Tarbiat Modares University (Tehran, Iran) Institutional Review Board (R.TMU.REC.1396.691).

### Sample-size calculation

In this proof-of-concept trial, we aimed to evaluate the potential intervention effect of MemFlex in a sample with PTSD relative to a wait-list control group. Because MemFlex has not been previously compared with a wait-list control, no prior studies were able to provide an anticipated between-groups effect size for use in a power calculation. Thus, we completed a power calculation to determine the sample size needed to detect a significant within-subjects improvement (if there was one) in memory flexibility, our primary cognitive target and proposed mechanism of change, from before intervention to after intervention in the MemFlex group on the basis of the prior trial of MemFlex for depression (Hitchcock et al., 2018). A power analysis using an estimated within-subjects improvement of *d* = 1.03 (two-tailed α = .05) suggested that 15 participants per group would provide 95% power. Because it is not desirable to use a within-subjects effect size to calculate power for a study testing between-groups effects, we also completed a power analysis using the estimated effect, *d* = 4.79, of an existing autobiographical memory-based intervention (MEST) on PTSD symptoms relative to a wait-list control (two-tailed α = .05; Moradi et al., 2014). This second power analysis indicated that eight participants per group would provide 99% power. To be as conservative as possible, we recruited the larger of the two suggested samples sizes and employed two-tailed null hypothesis tests when comparing the MemFlex and wait-list control conditions.

We had initially aimed to recruit 25 participants per group; however, recruitment needed to be ceased in February 2020 because of COVID-19 safety concerns. We were therefore required to close recruitment at 20 participants per group; however, because of our low 7% attrition rate, we did exceed the number estimated in our power calculation.

### Participants and recruitment

**Fig. 1.**
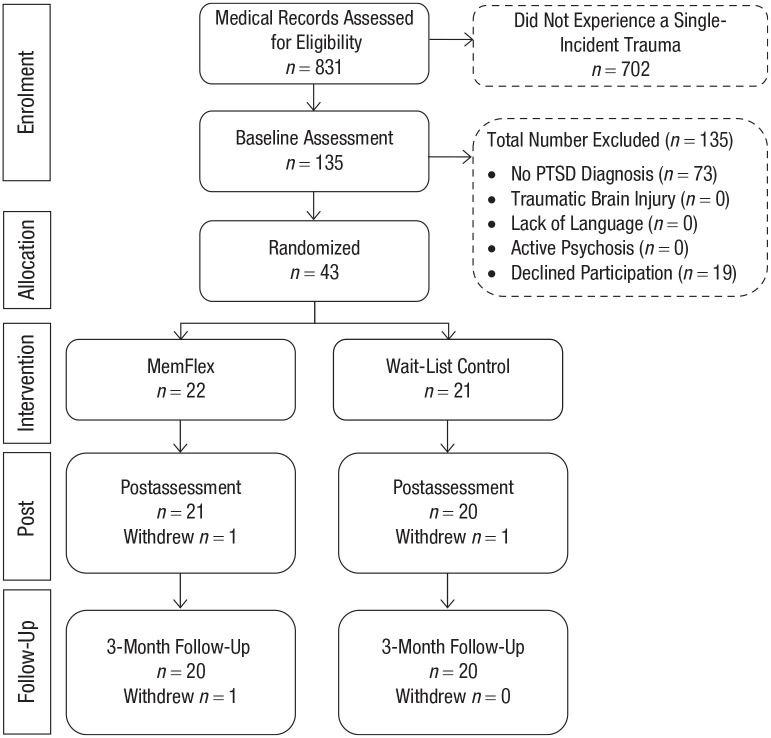
Consolidated Standards of Reporting Trials (CONSORT) diagram. All three participants who withdrew reported that they no longer wished to take part in the research when contacted to arrange assessments. PTSD = posttraumatic stress disorder.

The CONSORT diagram of study participation is presented in Figure 1. Forty-three adults (29 identifying as female) aged 21 to 43 years were recruited following presentation at the Sina Hospital in Tehran, Iran. Inclusion criteria were diagnosis of PTSD following the experience of a single-incident trauma (using the definition of trauma from the fifth edition of the *Diagnostic and Statistical Manual of Mental Disorders* [*DSM-5*]; American Psychiatric Association, 2013) and age over 18 years. All participants were seeking medical attention for injuries sustained in a road traffic accident that had occurred 3 to 18 months previously. PTSD diagnosis was determined by trained research staff using a Persian translation of the Structured Clinical Interview (SCID) for *DSM-5* (First et al., 2015) under the supervision of a clinical psychologist. All SCIDs were second rated by the clinical psychologist, who agreed on PTSD diagnosis for 100% of participants. Exclusion criteria were lack of fluency in Farsi, traumatic brain injury or cognitive impairment (indexed via self-report), and current experience of psychosis (determined via the SCID). Participants were able to continue with any concurrent psychological intervention or medication.

Potential participants were identified via trauma and injury details in hospital records and contacted by a researcher after discharge. After an initial telephone screening, participants meeting trauma type and age inclusion criteria were invited to a face-to-face appointment to complete the SCID. For participants meeting PTSD criteria on the SCID, a second appointment was booked for within the next week to deliver the baseline assessment and, if allocated to MemFlex, the workbook introduction. Allocation to condition was completed via a computer-generated random number allocation conducted by the trial statistician (P. Watson), who was blind to study objectives.

### Intervention

#### MemFlex

The MemFlex intervention aims to improve the ability to retrieve any autobiographical memory type on demand. Participants were never asked to retrieve negative memories or trauma memories. In particular, the intervention trains three core skills: balancing, elaboration, and flexibility. *Balancing* aims to improve access to personally relevant memories that are emotionally positive or benign. Improving ease of access to positive autobiographical information aimed to balance against the negative self-appraisals that drive PTSD. *Elaboration* aims to increase the detail and vividness of these positive memories. *Flexibility* explicitly trains the ability to move between specific and general levels of memory representation. These skills are trained via eight workbook-based sessions, which provide education about autobiographical memory distortions and the role of autobiographical memory in everyday life and cued recall tasks designed to provide practice with the three core skills. Participants are encouraged to complete two workbook sessions per week for 4 weeks and complete one face-to-face session with a researcher before beginning the workbook. In the face-to-face session, the rationale of the intervention is explained, the training exercises are introduced and practiced, and the researcher helps the participant to set a schedule for workbook completion. During workbook completion, participants can contact the researcher with any questions or concerns. Ninety-five percent of individuals contacted the researcher via text message to ask a question.

Two key changes were made to the MemFlex intervention evaluated by Hitchcock et al. (2018). First, the introductory session and Session 1 of the workbook were edited to reflect the impact of trauma (rather than depression) on autobiographical memory retrieval. The key concepts outlined were how the avoidance of the trauma memory (as a way to manage negative affect) can spread out to other memories not related to the trauma and how this can lead one to get stuck at the general, more abstract level of memory representation. Two pilot participants with PTSD completed this version of the workbook to ensure clarity and to provide lived experience input regarding how the workbook could be improved. Minor wording changes were made on the basis of feedback.

Second, the workbook was translated from English into Farsi in accordance with guidelines from the World Health Organization (n.d.). Adaptations were made to examples and pictures to make them culturally appropriate (e.g., an example of a visit to a pub was changed to a visit to a restaurant). The workbook was translated into Farsi by researchers familiar with the theories and psychological terms used in the workbook to ensure an accurate representation of the concepts being explained. A bilingual expert panel then reviewed the translation to identify inadequate expression and concepts. One concept (“tapestry,” which is used as a metaphor in the workbook) was identified as a potential mistranslation, and the principal investigators decided on the most appropriate terminology. The Farsi version was then back-translated into English by an independent translator with no experience with the subject matter. The back-translation was then read by the researchers, and inaccurate terminology or descriptions lacking in clarity were discussed with the translator. Researchers and translator then agreed on the final wording to be used.

#### Wait-list control

The wait-list control group completed the same number of face-to-face appointments. While the MemFlex participants were completing the workbook, the wait-list control group had no further contact. At the end of the 3-month follow-up assessment, wait-listed participants completed the introductory session for MemFlex. These participants than completed MemFlex over the following month, although further assessments were not administered.

### Measures

#### Cognitive outcome

Our primary cognitive outcome was the ability to retrieve either of two autobiographical memory types (specific, general) on demand, indexed by the total number of memories correctly recalled in an Alternating Instructions version of the Autobiographical Memory Task (AMT-AI; Dritschel et al., 2014). As in our prior use of this measure in an Iranian sample (Piltan et al., 2021), participants were required to retrieve a memory of their personal past in response to 24 Farsi cue words of positive, negative, and neutral emotional valence. Cues were matched between positive and negative valence for frequency and emotionality. A block of six cues requested retrieval of specific memories of single incident events, as in the original AMT (Williams & Broadbent, 1986); a block of six cues requested general memories that summarize categories of events, as in the Reversed version of the AMT (AMT-R; Dalgleish et al., 2007); and a block of 12 cues required the individual to alternate between retrieval of general and specific memories.

All task instructions were presented in written format on a computer, and examples of correct memory types were provided. Four practice trials (two each for specific and general memories) were completed, with feedback provided on incorrect responses, before the test trials. For each test trial, participants were presented with a screen requesting either a specific or general memory. This was immediately followed by a Farsi cue word presented individually on the screen, for which they were given 30 s to report a memory aloud. Responses were audio recorded and later coded as specific or general. Errors were responses scored as extended memories (i.e., event lasted longer than 1 day), repeated memories (i.e., a memory had been previously reported), a semantic associate (i.e., personal information that is related to the cue but is not a memory), or an omission (i.e., no response reported). Fifteen percent of AMT-AI responses were scored by a second rater, which demonstrated good interrater reliability, intraclass correlation coefficient = .96. As our primary outcome, we calculated the proportion of correct responses in which the number of omissions is subtracted from the number of presented cues, as in Piltan et al. (2021) and Hitchcock et al. (2018).

#### Primary clinical outcome

Our primary clinical outcome was score on the Persian version of the Posttraumatic Checklist for *DSM-5* (PCL-5; Jasbi et al., 2018). Our prior use of the Persian version of the PCL-5 demonstrated good internal consistency (Piltan et al., 2021), and Cronbach’s α was acceptable in the current sample, α = .79.

#### Secondary outcomes

We were also interested in the impact of MemFlex on other cognitive predictors of PTSD. Maladaptive trauma-related appraisals were measured using a Persian version of the Posttraumatic Cognitions Inventory (Foa et al., 1999). The self-report measure contains Likert-scale items for three subscales: negative cognitions about the world, negative cognitions about the self, and self-blame. Internal consistency was acceptable in the current sample, α = .81. A Persian translation of the Trauma Memory Quality Questionnaire (Meiser-Stedman et al., 2007) was also administered to determine whether training autobiographical memory retrieval more broadly may improve the trauma memory itself. Items index visual and sensory quality, temporal context, and the extent to which the memory is available in a verbally accessible format (cf. Brewin et al., 1996) because these are key memory features proposed to drive symptoms by leading cognitive models of PTSD (e.g., Ehlers & Clark, 2000). Internal consistency was acceptable in the current sample, α = .70. Finally, rumination was assessed via the Persian version of the Ruminative Response Scale (RRS; Treynor et al., 2003). The RRS consists of 22 items that index the tendency to ruminate in relation to sad mood. The scale yields both a total score and scores for three subscales: self-focus, symptom focus, and focus on possible causes and consequences of sad mood. Internal consistency for the total scale was acceptable in the current sample, α = .77.

#### Additional measures

To explore potential transfer to episodic simulation more broadly, we administered the Future Thinking Task. As in the AMT-AI, participants are provided with cue words but are asked to simulate a potential future event for five positive, five negative, and five neutral key words. The verbal-fluency task (VFT; Spreen & Strauss, 1998) indexes executive control over verbal information. In the VFT, participants are given 60 s to generate as many words as they can beginning with a certain letter. Another 60 s are then given to generate as many words as possible that fit a given semantic category (e.g., “animals”). Total score was the number of correctly identified words in each condition minus errors (errors were repeated words, proper nouns, and words that do not fit the given instructions). Given prior effects of MemFlex on depression and comorbidity between PTSD and depression, the Farsi version of the Beck Depression Inventory–II was also administered. The Farsi version of the BDI-II demonstrates good reliability and validity (Ghassemzadeh et al., 2005). Internal consistency was acceptable in the current sample, α = .75.

### Procedure

Written informed consent was provided before beginning the SCID. The preintervention and postintervention assessments consisted of all measures. In the initial session, participants were instructed to complete two MemFlex sessions each week for 4 weeks. At the end of the 4 weeks, the researcher contacted the participant to check that the workbook had been completed and to book the postintervention assessment. There was a mean of 31.7 days (*SD* = 1.86) between preintervention and postintervention assessments (range = 28–36), and this did not differ between conditions, *t*(38) = 0.51, *p* = .62. Three months after the postintervention assessment, the follow-up assessment administered all measures with the exception of the VFT because of lack of parallel versions. There was a mean of 92.4 days (*SD* = 4.52) between postintervention and follow-up assessments (range = 84–103), and again this did not differ between conditions, *t*(38) = 0.55, *p* = .58. Stimuli for the AMT-AI, Future Thinking Task, and VFT were counterbalanced between assessments. Blinded assessors administered and scored all assessments, which were completed face-to-face at the university or hospital, depending on participant preference. Any arising clinical risk issues were managed by the supervising clinical psychologist. No adverse events were reported during this trial. Participants were reimbursed the equivalent of $20 for their time.

## Results

### Sample and intervention characteristics

Baseline characteristics are presented in Table 1. Groups did not significantly differ in any demographic or clinical characteristics, *p*s > .14. All participants identified as Asian in ethnicity. No participants received psychological or pharmacological treatment for mental health issues over the course of the study.

**Table 1.** Sample Characteristics

Characteristic	Group	
	MemFlex (*n* = 22)	Wait-list (*n* = 21)
Female (*n*)	12	17
Age	29.00 (5.84)	29.76 (6.04)
Completed secondary school (*n*)	7	6
Completed postgraduate or undergraduate degree (*n*)	15	14
Currently employed or engaged in full-time studies	15	17
Verbal-fluency task^a^	24.59 (2.95)	25.62 (4.79)
Posttraumatic Checklist–5^b^	44.09 (9.98)	43.67 (9.74)
Beck Depression Inventory–II^c^	25.45 (7.51)	21.24* (6.15)
Comorbid anxiety disorder (*n*)	0	2
Comorbid major depressive disorder (*n*)	6	2

Note: Values are means with standard deviations in parentheses unless otherwise specified. Highest level of obtained education is displayed. In the wait-list control group, one participant had completed only primary school and is therefore not displayed.

a Spreen & Strauss (1998). ^b^Jasbi et al. (2018). ^c^Ghassemzadeh et al. (2005).

* *p* = .051.

Across both conditions, we experienced low (7%) attrition for assessments, losing two participants after the intervention and one at follow-up. We achieved excellent adherence for workbook completion. In the MemFlex group, 100% of participants completed all eight sessions (operationalized as having attempted each exercise in the session).

### Analysis of primary and secondary outcomes

An independent statistician (P. Watson) determined between-groups differences in all outcomes after intervention and follow-up, covarying for baseline scores (as per recommendations of Clifton & Clifton, 2019). Because the percentage of missing data values (3%) was lower than 5%, complete case analysis was used rather than the planned multiple imputation, as per the recommendations of Jakobsen et al. (2017). Because this is a proof-of-concept trial with a small sample size, consideration of potential treatment efficacy should be based on effect sizes and associated confidence intervals (CIs). Cohen’s *d* was calculated from the *F* value of relevant analyses and is reported with 95% confidence intervals.

### Primary cognitive target

**Fig. 2.**
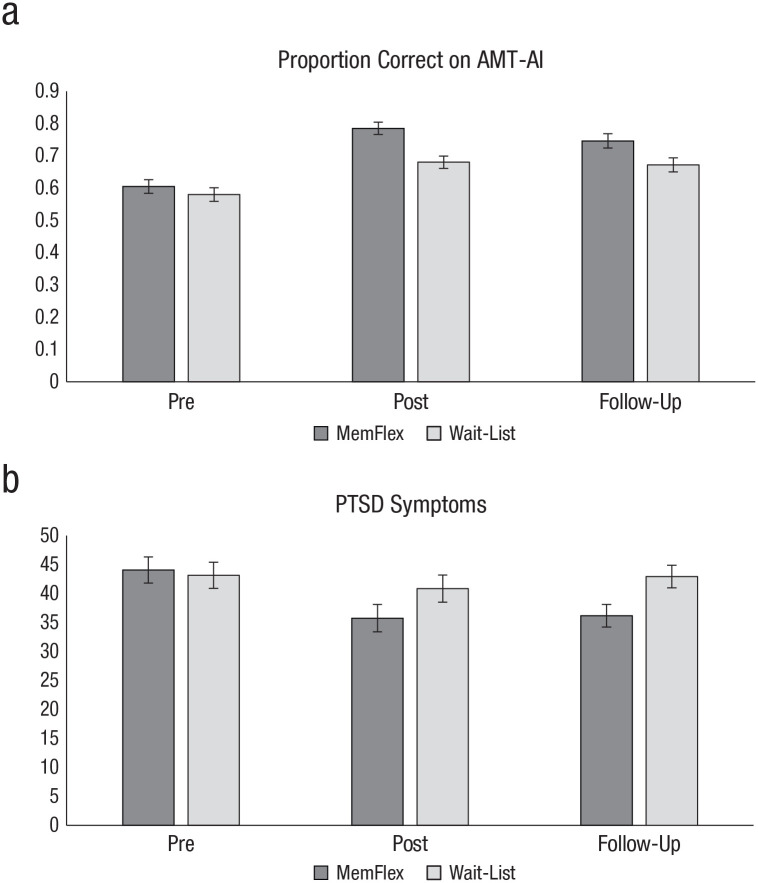
Mean (standard error) performance on (a) the primary cognitive outcome, the Alternating Instructions version of the Autobiographical Memory Test (AMT-AI), and (b) the primary clinical outcome, posttraumatic stress disorder (PTSD) symptom scores on the Posttraumatic Checklist-5, by group and assessment point. For the raw data, see the Supplemental Material.

As hypothesized, the MemFlex group demonstrated a higher proportion of correct responses on the AMT-AI at postintervention relative to the wait-list control group, *F*(1, 38) = 11.88, *p* = .001, *d* = 1.09, 95% CI = [0.40, 1.78]; the effect size for the difference was reduced slightly at 3-month follow-up, *F*(1, 37) = 5.05, *p* = .03, *d* = 0.70, 95% CI = [0.03, 1.37]. A significant Time × Allocation interaction, *F*(2, 37) = 3.25, *p* = .044, *d* = 0.57, 95% CI = [0.09, 1.23], indicated that although all participants improved from baseline to follow-up, *F*(2, 37) = 44.14, *p* < .001, *d* = 2.10, 95% CI = [1.28, 2.92], the degree of improvement was larger in the MemFlex group (*d* = 2.16, 95% CI = [1.33, 2.98]) relative to wait-list (*d* = 0.98, 95% CI = [0.30, 1.67]), *F*(1, 38) = 9.13, *p* = .004 (see Fig. 2).

Although there were no significant differences in baseline clinical characteristics between the two groups, there were more participants with comorbid major depressive disorder (MDD) in the MemFlex group relative to the wait-list control group. Thus, we repeated analysis of the cognitive target when covarying for comorbid MDD. Effects remained significant and of a similar effect size when comorbid MDD was covaried: at postintervention, *F*(1, 37) = 13.12, *p* = .001, *d* = 0.69, 95% CI = [0.03, 1.35]; at follow-up, *F*(1, 36) = 4.75, *p* = .036, *d* = 1.13, 95% CI = [0.43, 1.83].

To explore whether effects were driven by performance on a particular block (i.e., retrieval of specific memories, general memories, or alternating between the two) of the AMT-AI, we completed a follow-up Group × Block analysis of covariance (covarying baseline scores) predicting the proportion of correct responses. The Group × Block interaction was nonsignificant and of small effect size both at postintervention, *F*(2, 72) = 0.63, *p* = .53, *d* = 0.25, 95% CI = [−0.39, 0.89], and at follow-up, *F*(2, 70) = 0.83, *p* = .44, *d* = 0.29, 95% CI = [−0.36, 0.94], suggesting that the MemFlex group outperformed the wait-list group on specific, general, and alternating blocks of the AMT-AI.

### Primary clinical outcome

Effect sizes in favor of the MemFlex group, relative to the wait-list control group, were also observed on PTSD symptoms. Although a moderate, nonsignificant between-groups effect size was observed at postintervention, *F*(1, 38) = 2.59, *p* = .12, *d* = 0.50, 95% CI = [−0.15, 1.15], the MemFlex group achieved significantly lower PCL-5 scores at follow-up relative to the wait-list control group, with a large effect size, *F*(1, 37) = 9.17, *p* = .004, *d* = 0.96, 95% CI = [0.27, 1.65]. Exploratory analyses of the PCL-5 subscales are presented in the Supplemental Material available online.

We next calculated the percentage of participants who experienced clinically meaningful change on the PCL-5 using guidelines for the PCL-4, as per the recommendations of the U.S. National Center for PTSD (change values have not yet been determined for the PCL-5 but are expected to be similar to PCL-4; Weathers et al., 2013). Of participants completing MemFlex, 70% demonstrated a response to treatment (i.e., “reliable change,” indexed as a ≥ 5-point improvement on the PCL-5 by follow-up; Weathers et al., 2013), compared with 40% in the wait-list control group, χ^2^(1) = 3.64, *p* = .057. Forty percent of participants in the MemFlex group demonstrated “clinically significant change” on the PCL-5 (indexed as a ≥ 10-point improvement on the PCL-5 by follow-up; Weathers et al., 2013), relative to 15% of wait-list participants, χ^2^(1) = 3.13, *p* = .077.

### Secondary outcomes

On other cognitive predictors of PTSD, a significant, moderate effect size was found in favor of the MemFlex group, relative to the wait-list control group, for posttraumatic cognitions at postintervention, *F*(1, 38) = 5.38, *p* = .026, *d* = 0.72, 95% CI = [0.06, 1.38]. This increased to a large effect size at follow-up, *F*(1, 37) = 7.23, *p* = .011, *d* = 0.85, 95% CI = [0.17, 1.53]. Although nonsignificant effects were found on trauma memory quality, the moderate effect sizes observed were in favor of the MemFlex group both at postintervention, *F*(1, 38) = 1.89, *p* = .18, *d* = 0.43, 95% CI = [−0.22, 1.08], and at follow-up, *F*(1, 37) = 3.46, *p* = .07, *d* = 0.59, 95% CI = [−0.07, 1.25].

Although a negligible between-groups effect size was observed for rumination at posttreatment, *F*(1, 38) = 0.33, *p* = .57, *d* = 0.18, 95% CI = [−0.46, 0.82], the effect size at follow-up suggested that the MemFlex group might experience more rumination compared with the wait-list control group, *F*(1, 37) = 3.22, *p* = .08, *d* = 0.57, 95% CI = [−0.09, 1.23]. This appeared to be driven by a decrease in rumination in the wait-list control group from postintervention to follow-up, *t*(19) = 2.79, *p* = .012, whereas the MemFlex group did not significantly change, *t*(19) = 0.81, *p* = .43.

### Additional measures

In terms of transfer to broader episodic simulation skills, there was minimal evidence for an effect on the total number of future specific events generated either at postintervention, *F*(1, 38) = 0.74, *p* = .39, *d* = 0.27, 95% CI = [−0.37, 0.91], or at follow-up, *F*(1, 37) = 0.84, *p* = .37, *d* = 0.29, 95% CI = [−0.36, 0.94]. A large, significant effect in favor of the MemFlex group, relative to the wait-list control group, was observed on depressive symptoms at postintervention, *F*(1, 38) = 6.51, *p* = .015, *d* = 0.80, 95% CI = [0.13, 1.47], but this was not maintained at follow-up, *F*(1, 37) = 0.39, *p* = .54, *d* = 0.20, 95% CI = [−0.45, 0.85].

### Exploratory analysis

Finally, we completed a parallel mediation model in PROCESS using 10,000 bias-corrected bootstrapped samples to explore whether change in autobiographical memory retrieval or posttraumatic cognitions mediated the effect of MemFlex on PTSD symptoms. We predicted the effect of intervention condition on PCL-5 at follow-up, covarying for baseline PCL-5 score, with preintervention to postintervention change in AMT-AI performance and PTCI entered as parallel mediators. The overall model was significant, *F*(2, 37) = 11.11, *p* < .001. Although both baseline PTSD symptoms, *b* = 0.45, *SE* = 0.12, *p* = .006, and intervention, *b* = 7.21, *SE* = 2.38, *p* = .005, significantly predicted later symptoms, no evidence was found for indirect effects of intervention via change in AMT-AI performance, *b* = −0.43, *SE* = 1.28, 95% CI = [−2.70, 2.31], or posttraumatic cognitions, *b* = 1.87, *SE* = 1.43, 95% CI = [−0.08, 6.41].

We also explored whether individual blocks on the AMT-AI mediated intervention effects. We predicted the effect of intervention condition on PCL-5 at follow-up, covarying for baseline PCL-5 score, with preintervention to postintervention change in AMT-AI performance in the specific, categoric, and alternating blocks as parallel mediators. Small effect sizes were observed for the indirect effect via specific blocks, *b* = 0.89, *SE* = 1.23, 95% CI = [−0.77, 4.44]; categoric blocks, *b* = 0.12, *SE* = 0.98, 95% CI = [−1.36, 2.85]; and alternating blocks, *b* = −1.04, *SE* = 1.09, 95% CI = [−4.60, 0.35], although variation in the direction and size of the effects suggests this may warrant detailed investigation in a fully powered trial.

## Discussion

This proof-of-concept randomized controlled trial evaluated a low-intensity, basic-science-driven intervention that sought to target a cognitive predictor of PTSD—autobiographical memory retrieval—to drive a reduction in PTSD symptoms. We provided initial support for use of MemFlex for improving both autobiographical memory flexibility and PTSD symptoms, relative to a wait-list control, in a diagnosed sample. Results also suggested a significant, moderate to large effect size in favor of MemFlex on maladaptive posttraumatic cognitions and a moderate, although nonsignificant effect on trauma memory quality—both of which are key cognitive mechanisms underlying PTSD prognosis that are targeted in high-intensity evidence-based treatments for PTSD. Now that we have established an effect on the proposed mechanism of action of MemFlex, with corollary effects on symptom scores (Craig et al., 2008; Medical Research Council, 2000), a fully powered efficacy trial comparing MemFlex with another low-intensity intervention is warranted.

A key motivation for using MemFlex for PTSD is an attempt to ameliorate cognitive-based predictors of symptoms, particularly memory factors, as occurs in the most effective higher-intensity interventions for PTSD (e.g., trauma-focused CBT, EMDR). First, results suggested a moderate to large effect on voluntary retrieval of specific memories and the ability to move between specific and general levels of memory representation. Significant improvement over time in the wait-list control group indicates that practice effects are likely on the AMT-AI; however, the degree of improvement was much larger in participants completing MemFlex. Although autobiographical memory retrieval has been established as a direct predictor of prognosis, improved memory flexibility may also aid the ability to retrieve the most useful type of memory to be used in service of other cognitive skills. For example, when solving an interpersonal problem, a general memory of “when we fight, it’s usually best if I give my partner some space before we talk” may be more useful than a specific memory (e.g., “our last argument was about my drinking”). Because episodic simulation is proposed to require bridging of abstract and concrete levels of representation, we did anticipate that increased ability to move flexibly between levels of abstraction may support improved episodic simulation (Zhao et al., 2020). However, we did not find evidence of this. An important feature of a scaled-up study will be to further elucidate the mechanisms through which increased memory flexibility may improve PTSD symptoms and to explore whether particular subtypes of PTSD symptoms (e.g., reexperiencing, negative cognitions) are differentially affected by the intervention.

Second, promising effect sizes were found for other cognitive predictors of PTSD that are explicitly targeted in higher-intensity treatments. Assisting participants to move away from broader memory-based generalizations of the self appeared to help to reduce overgeneralized, maladaptive cognitions that drive PTSD (e.g., I am a weak person; I used to be a happy person, but now I am always miserable). This is consistent with our prior experimental finding that autobiographical memories help to appropriately restrain generalized, negative self-beliefs (Hitchcock et al., 2017). Likewise, our results did suggest that an autobiographical memory-based intervention may be able to improve the quality of a trauma memory without directly targeting that memory. Although this has interesting implications for theory and for extracting active components of higher-intensity treatments into low-intensity treatment options, the moderate to large effect size was nonsignificant, as was our evaluation of mediation effects, and this will need to be evaluated in a later trial. In addition, we used a self-report measure of trauma memory quality, and objective ratings of trauma narratives in the future might provide a more rigorous evaluation of the impact of MemFlex on trauma memories.

Our results suggest it may take some time for any immediate improvements in autobiographical memory to translate to improvements in PTSD symptoms given that stronger effects were observed at follow-up. There is considerable variability in the effects of other autobiographical memory-based interventions on posttraumatic stress (e.g., MEST; Moradi et al., 2014), with meta-analysis suggesting that any effects may be immediate and not maintained at follow-up (see Barry et al., 2019). Contrary to prior evaluations of MemFlex (but similar to prior evaluations of MEST for PTSD; Moradi et al., 2014), the treatment effect on depressive symptoms was not maintained at follow-up, although it is worth noting that the overall comorbidity of depression in our sample (18.6%) was lower than commonly observed in PTSD. Although the PTSD symptom endpoint still indicated probable PTSD (above 31 on the PCL-5), it is impressive that a low-intensity intervention that does not involve working with trauma-related cognitions or PTSD symptoms at all was able to produce a reliable change on our measure of PTSD symptoms (as per Weathers et al., 2013) for 70% of participants. This now needs to be evaluated with more varied and severe trauma types because it is unclear whether such findings would hold in people with more interpersonally focused or prolonged traumatic experiences. Our sample was also relatively well educated, thus future samples should also aim to represent more diverse education experiences.

Although it is the recommended first step in development of complex interventions (Medical Research Council, 2000), the wait-list control design of this study has inherent limitations. Future research will need to evaluate MemFlex against an active intervention to determine whether demand or expectancy effects influence the treatment effects observed in this study. Future research may also wish to explore session-to-session change in the targeted memory skills. Key benefits of the MemFlex intervention include that it is not reliant on access to a computer or the Internet and so may be easily disseminable within low- and middle-income countries or for people in rural areas. Although the MemFlex intervention was developed in an English-speaking Western setting, our study provides initial support for the efficacy of the intervention in a Middle Eastern cultural setting. Autobiographical memory deficits have been observed across a variety of languages and cultures (e.g., Jobson et al., 2016, 2018), thus the intervention may be suitable for translation into different languages. Because the initial face-to-face session has now been effectively delivered by undergraduate and postgraduate students without any counseling or therapeutic training, the intervention could lend itself well to delivery by nonexperts (e.g., teachers, other health practitioners, minimally trained volunteers). In the future, it may be worth exploring whether this face-to-face session could be replaced by an additional workbook session or potentially a video to allow flexibility in delivery. Because these applications may help to improve access to cognitive-based intervention for PTSD that is low cost, low intensity, focused on the individual’s positive experiences, and able to be facilitated by nonexperts, a scaled-up trial of MemFlex is now needed to evaluate treatment efficacy and the mechanisms driving any treatment effects.

## Supplemental Material

sj-pdf-1-cpx-10.1177_2167702620982576 – Supplemental material for Proof of Concept for the Autobiographical Memory Flexibility (MemFlex) Intervention for Posttraumatic Stress Disorder Click here for additional data file. Supplemental material, sj-pdf-1-cpx-10.1177_2167702620982576 for Proof of Concept for the Autobiographical Memory Flexibility (MemFlex) Intervention for Posttraumatic Stress Disorder by Ali Reza Moradi, Maryam Piltan, Mohammad Hasan Choobin, Parviz Azadfallah, Peter Watson, Tim Dalgleish and Caitlin Hitchcock in Clinical Psychological Science

## Transparency

*Action Editor:* Christopher G. Beevers

*Editor:* Kenneth J. Sher

*Author Contributions*

T. Dalgleish and C. Hitchcock are joint senior authors. C. Hitchcock, A. R. Moradi, and M. Piltan conceived the study. T. Dalgleish provided expert guidance on study methods. M. Piltan, M. H. Choobin, and P. Azadfallah completed data collection. P. Watson completed data analysis. All of the authors contributed to writing the manuscript and approved the final manuscript for submission.

**Open Practices:** All data have been made publicly available via OSF and can be accessed at https://osf.io/u2hqf. The design and analysis plans for the experiments were preregistered at https://clinicaltrials.gov/ct2/show/NCT03634709. This article has received badges for Open Data and Preregistration. More information about the Open Practices badges can be found at https://www.psychologicalscience.org/publications/badges.
