# Supplementary material for: Proof of Concept for the Autobiographical Memory Flexibility (MemFlex) Intervention for Posttraumatic Stress Disorder
Source: Clin Psychol Sci. 2021 Mar 31;9(4):686–98. doi: 10.1177/2167702620982576 (PMC8278548; doi:10.1177/2167702620982576)
Supplement: sj-pdf-1-cpx-10.1177_2167702620982576 – Supplemental material for Proof of Concept for the Autobiographical Memory Flexibility (MemFlex) Intervention for Posttraumatic Stress Disorder [file sj-pdf-1-cpx-10.1177_2167702620982576.pdf]

SUPPLEMENTAL MATERIAL

Proof-of-concept for the autobiographical Memory Flexibility (MemFlex) intervention for Posttraumatic Stress Disorder

Ali Reza Moradi[1]

Maryam Piltan[2]

Mohammad Hasan Choobin[3]

Parviz Azadfallah[2]

Peter Watson[4]

Tim Dalgleish[4, 5+]

Caitlin Hitchcock[4+ *]

[1]Department of Psychology, Kharazmi University, Institute for Cognitive Sciences Studies

[2] Department of Psychology, Tarbiat Modarres University

[3] Department of Clinical Psychology, Kharazmi University

[4] MRC Cognition and Brain Sciences Unit, University of Cambridge

[5] Cambridgeshire and Peterborough NHS Foundation Trust

+ joint senior authors

* corresponding author; Caitlin.hitchcock@mrc-cbu.cam.ac.uk, +44 1223 273 744,

15 Chaucer Road Cambridge CB2 7EF, United Kingdom

Funding: This project was funded by the British Academy (SG162207). Caitlin Hitchcock was partly supported by the Economic and Social Research Council (ES/R010781/1). Tim Dalgleish was supported by the Medical Research Council (SUAG/043 G101400).

Table S1. Mean (standard deviation) score on the cognitive target and primary clinical outcome at each assessment, by intervention condition.

	MemFlex			Waitlist		
	Pre	Post	Follow-up	Pre	Post	Follow-up
AMT-AI						
Specific	.58 (.12)	.73 (.15)	.70 (.15)	.61 (.16)	.60 (.10)	.64 (.15)
Categoric	.70 (.15)	.76 (.15)	.75 (.12)	.62 (.14)	.66 (.20)	.72 (.15)
Alternating	.57 (.14)	.78 (.10)	.75 (.09)	.54 (12)	.69 (.13)	.66 (.13)
Total	.61 (.10)	.78 (.08)	.75 (.08)	.58 (.08)	.68 (.09)	.67 (.11)
PCL-5						
Intrusions	10.91 (3.54)	8.67 (3.61)	8.25 (3.34)	11.67 (2.95)	10.45 (3.84)	11.05 (1.88)
Avoidance	4.32 (1.76)	3.62 (1.63)	3.70 (1.59)	4.95 (1.46)	4.60 (2.19)	4.70 (1.59)
Negative cognitions	14.64 (3.54)	12.29 (3.58)	12.40 (3.73)	13.05 (4.07)	13.45 (4.50)	14.15 (4.52)
Hyperarousal	14.14 (3.74)	11.52 (3.54)	11.80 (3.04)	14.00 (3.86)	12.35 (4.29)	13.05 (3.50)
Total	44.09 (9.98)	36.09 (10.00)	36.15 (9.61)	43.67 (9.74)	40.85 (10.96)	42.95 (7.71)

Note. AMT-AI= Alternating Instructions Autobiographical Memory Test; Categoric = Categoric block of AMT-AI; Specific= Specific block of AMT-AI; Alternating = Alternating block of AMT-AI; PCL-5=Posttraumatic Checklist-5. There was a moderate effect size for the condition × time interaction on PCL-5, $F(2, 76)=3.46$, $p=.036$, $d=0.59$ [0.07, 1.25], and a small-moderate effect size for the condition × time × PCL-5 subscale interaction, $F(6, 228)=1.89$, $p=.08$, $d=0.43$ [-0.22, 1.09]. There was a a moderate effect size for the condition × time interaction on AMT-AI scores, $F(2, 76)=3.46$ $p=.037$, $d=0.59$ [0.07, 1.25], and small-moderate effect size for the condition × time × AMT-AI block interaction, $F(4, 152)=1.87$, $p=.12$, $d=0.43$ [-0.22, 1.09].

Table S2. Pearson correlations (n=40) between pre-to-post intervention change in the cognitive target and clinical outcomes.

	1.	2.	3.	4.	5.
1. AMT-AI					
2. Specific	.484**				
3. Categoric	.685**	.496			
4. Alternating	.786**	-.003	.447*		
5. PCL-5	-.253	-.313*	-.311*	.009	
6. BDI-II	-.312*	-.439*	-.204	-.025	.412*

Note. * $p<.05$ ** $p\leq.001$. AMT-AI=Total score on Alternating Instructions Autobiographical Memory Test; Categoric = Categoric block of MAT-AI; Specific= Specific block of AMT-AI; Alternating = Alternating block of AMT-AI; PCL-5=Posttraumatic Checklist-5; BDI-II = Beck Depression Inventory II.
